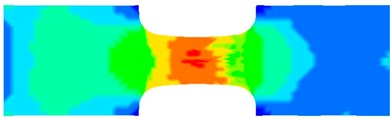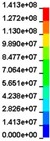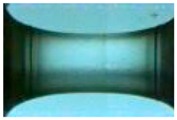# Experimentation and Modeling of the Tension Behavior of Polycarbonate at High Strain Rates

**DOI:** 10.3390/polym8030063

**Published:** 2016-02-29

**Authors:** Yingjie Xu, Tenglong Gao, Jun Wang, Weihong Zhang

**Affiliations:** 1 Engineering Simulation and Aerospace Computing (ESAC), Northwestern Polytechnical University, Xi’an 710072, China; gaotl@mail.nwpu.edu.cn; 2 Mechanics Unit (UME), ENSTA-ParisTech, Paris, 91761 Palaiseau, France; jun.wang@ensta-paristech.fr

**Keywords:** polycarbonate, tension, high strain rate, constitutive model

## Abstract

A comprehensive understanding of the mechanical behavior of polycarbonate (PC) under high-rate loadings is essential for better design of PC products. In this work, the mechanical behavior of PC is studied during tensile loading at high strain rates, using a split Hopkinson tension bar (SHTB). A modified experimental technique based on the SHTB is proposed to perform the tension testing on PC at rates exceeding 1000 s^−1^. The effect of strain rates on the tension stress–strain law of PC is investigated over a wide range of strain rates (0.0005–4500 s^−1^). Based on the experiments, a physically based constitutive model is developed to describe the strain rate dependent tensile stress–strain law. The high rate tensile deformation mechanics of PC are further studied via finite element simulations using the LSDYNA code together with the developed constitutive model.

## 1. Introduction

Polycarbonate (PC) is a thermoplastic polymeric material with high transparency, high ductility, impact resistance and is comparatively lightweight. It has been widely used in impact protection applications including aircraft canopies, face shields, goggles, windshields and windows and blast shields [1,2]. Thus, impact response of the PC products is a subject of critical interest. High strain rates are commonly encountered during an impact event such as projectile striking or blast loading. Therefore, an accurate understanding and modeling of the mechanical behavior of PC at high strain rates is of great importance.

The mechanical behavior of materials at high strain rates has been extensively studied using various experimental procedures ranging from impact (gas gun impact, Taylor impact, and Izod impact) tests [3] to split Hopkinson pressure bar (SHPB) test [4]. Amongst these procedures, SHPB test has been instrumental in obtaining stress–strain behavior of materials at high strain rates up to 10^3^ s^−1^.

Recently, experimental studies with SHPB have been performed to investigate the compressive behavior of PC at high strain rates [5,6,7,8,9,10,11]. Compared with metals, high-rate experiment of PC in compression with the SHPB is more complicated due to its low density, low modulus and low yield stress. The low densities and low elastic wave velocities ultimately result in low impedances, which lead to low amplitude of transmitted pulses and consequently increase the signal-to-noise ratio. To address these problems, some modifications such as the pulse shaping technique and the use of low-impendence aluminum, titanium or polymeric bars are proposed to perform the SHPB test [12]. Experimental results indicate that PC exhibits an obvious elastic-plastic stress–strain behavior, including linear elasticity, nonlinear elasticity, yielding, post-yield strain softening and strain hardening when subjected to high strain rates compressive loading. Moreover, it can be observed that the yield stress increases dramatically as the strain rate increases. Mulliken and Boyce [8] investigated the relationship between the yield stress and logarithm strain rate in a wide strain-rate region and indicated that there exists a transition threshold of rate sensitivity for yield behavior of PC.

Owing to the fact that there exist significant differences in tensile and compressive deformation behavior of polymers under high strain rate loadings [13], it is therefore necessary to study the dynamic tension behavior of PC. Moreover, tension tests provide more understandings on damage, failure and fracture behavior of materials. However, due to the experimental difficulties in tensile test with SHPB, very few studies have been reported on the tension behavior for polymers [14,15,16,17,18], especially for PC [17,18] at high strain rates. Design change of the SHPB into a split Hopkinson tension bar (SHTB) enabling tensile loading conditions is thus required for the high strain rate tensile test. Sarva and Boyce [17] used a split-collar type SHTB to investigate the behavior of PC during tensile loading at high strain rates. In their study, the threaded PC sample was attached between two bars and a split collar surrounding the sample was sandwiched between two bars. By using the split collar, the initial compressive pulse was transmitted into the second bar without loading the sample. The subsequent reflected tensile pulse (from the free rear end of the second bar) then loaded the sample. By using this split-collar type SHTB, the effects of varying strain rate, overall imposed strain magnitude and specimen geometry on the mechanical response were examined in details. Cao *et al.* [18] implemented high strain rate tension tests of PC using a modified SHTB. The PC sample was bonded to incident and transmitted bars by means of adhesion. The incident stress pulse was initiated by the impact of the hammer fixed on the high-speed rotating disk on the impact block, which caused the prefixed metal bar connected to the block and the incident bar to deform. The deformation of the prefixed metal bar generated the tensile loading stress pulse.

A constitutive model that accurately represents the material response of PC can provide convenient and useful guidelines on product design and effectively decrease the experimental cost. Significant advances have been achieved in developing phenomenological models and physically based models to describe the large strain, temperature, and rate-dependent deformation behavior of PC and other polymeric materials. Mulliken and Boyce [8] proposed a physically based high-rate constitutive model for the three-dimensional deformation of PC and Poly (methyl methacrylate) (PMMA) at strain rates ranging from 10^−4^ to 10^4^ s^−1^. Uniaxial compression tests were used to characterize the rate-dependent yield and post-yield behavior of PC and PMMA. The proposed constitutive model was shown to correctly predict yield stress values, as well as the strain rate regime of the transition in the yield behavior. Sarva and Boyce [17] then extended this constitutive model to encompass high-rate tensile behavior of PC. Anand *et al.* [19] conducted large-strain compression experiments on PC and PMMA and presented a thermo-mechanically coupled elasto-viscoplasticity model to describe the strain rate and temperature dependent large-deformation response. Based on the high-rate tensile experiment investigation, Cao *et al.* [18] developed a physically based three-dimensional elastic-plastic constitutive model to characterize the rate-temperature dependent yield and post-yield behavior of PC when subjected to tension loading. Bouvard *et al.* [20] presented a constitutive model for amorphous polymers using a thermodynamic approach with physically motivated internal state variables. Model parameters were determined for PC, PMMA and polystyrene using high-rate compressive test results. Based on an elastic-viscoplastic rheological approach, Richeton *et al.* [21] developed a three-dimensional constitutive model to describe the material response of PC and PMMA for a large range of strain rates. The model parameters are fitted for each polymer on the experimental data of high-rate uniaxial compression tests. The aforementioned models have shown their abilities to capture the mechanical response of PC within a large strain rate range. However, these models are primarily calibrated using the results of high strain rate compressive data. Thus, most of the models can be only used to predict the high strain rate compressive behavior of PC.

From the literature review it can be concluded that characterizing the tensile behavior of PC at high strain rates is still insufficient at present, in both experimentation and modeling. Most of existed studies of the high rate deformation of PC concern the compressive behaviors. Due to the experimental difficulties in tensile test with SHPB, very few studies have been reported on the high rate (exceeding 1000 s^−1^) tensile behavior for PC. This is why we conduct a detailed investigation of the tensile behavior of PC at high strain rates in the present paper. A modified experimental technique based on the SHTB is proposed to perform the tension testing on PC at rates exceeding 1000 s^−1^. The chronological progression of the dynamic deformation is captured with a high-speed CCD camera. Quasi-static tensile tests are conducted as well to investigate the effects of varying strain rate on the mechanical response of PC. Based on the experimental investigation, a rate dependent constitutive model is developed to describe the strain-rate dependent tension stress–strain law. The high rate tensile deformation mechanics of PC are further studied via finite element simulations using the LSDYNA code together with the developed constitutive model.

## 2. Experiment

### 2.1. Material and Specimen

**Figure 1:**
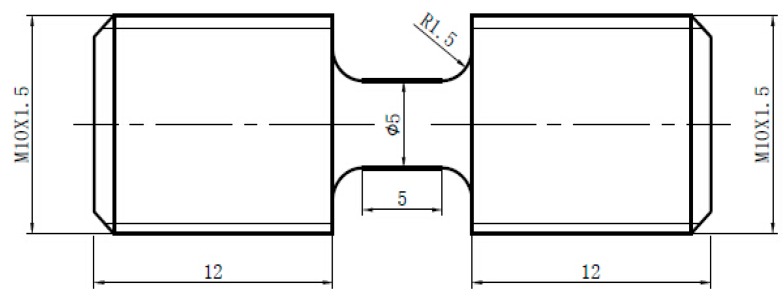
Geometry and dimension of the specimen for SHTB test.

The material used in the present study is PC Lexan 141R. The mass density of PC is 1.2 × 10^3^ kg/m^3^. The glass transition temperature (*T*_g_) of PC determined by the Dynamic Mechanical Thermal Analysis (DMTA) is 148 °C. Geometry and dimension of the specimen used for both SHTB and quasi-static tensile tests are shown in Figure 1. Notice that tight-handed and left-handed threads are provided on either side of these specimens for easy mounting. All the specimens are carefully manufactured from 12 mm thick plates of PC and kept at room temperature for more than three days prior to testing.

### 2.2. High Strain-Rate Uniaxial Tension Test

**Figure 2:**
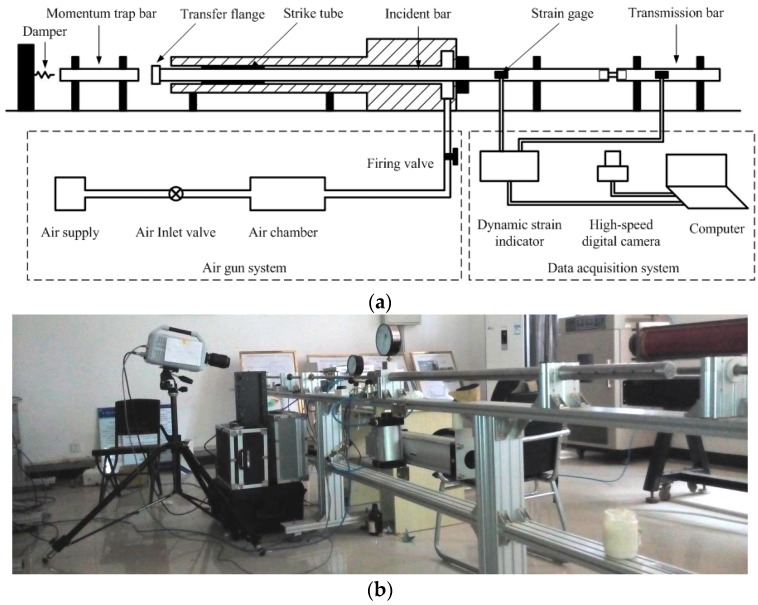
The SHTB test system: (**a**) schematic diagram; and (**b**) photograph.

High strain-rate tension tests at 10^3^ s^−1^ strain rate are carried out using the SHTB shown in Figure 2. The SHTB setup consists of an air gun system, a striker tube, an incident bar with a transfer flange at one end, a transmission bar, a momentum trap bar and the data acquisition system. The striker tube and all the bars are made of 18Ni steel and behave elastically during the test. The striker tube is 380 mm in length, with an outer diameter of 26 mm and inner diameter of 19 mm. The incident and transmission tension bars are 2800 and 1200 mm in length, respectively; both are 19 mm in diameter. The momentum trap bar is 430 mm in length and 19 mm in diameter. Both the incident bar and transmission bar contain threaded holes to connect the specimen. The air gun system is used to propel the striker tube. A detailed illustration of the working principle of the air gun system can be found in [22].

The striker tube accelerated by the air gun slides along the incident bar and impacts the flange to generate a tensile pulse in the incident bar. The duration of the incident pulse can be controlled by adjusting the length of the striker tube. As shown in Figure 2, a gap is precisely preset to separate the momentum trap bar and the transfer flange of the incident bar. This gap is set such that the end of the momentum trap bar and the face of the transfer flange are brought in contact, once the tensile pulse generated by the striker tube is completely transferred into the incident bar through the transfer flange. The generated tensile pulse travels down the incident bar and then propagates into the specimen, where it is partly transmitted into the transmission bar, and is partly reflected as a compressive pulse back into the incident bar. This reflected compressive pulse is then transmitted into the momentum trap bar, and reflects off the free end of this bar as a tensile pulse. Since the contact interface with the transfer flange cannot support tension, this tensile pulse is trapped in the momentum trap bar. Therefore, the reloading of the specimen by the reflected pulse is effectively prevented.

The theoretical value of the separation between the momentum trap bar and the transfer flange, *u_s_*, can be estimated by the following expression:

(1) $$u_{s}=2C_{0}\int_{0}^{t}\varepsilon_{c}\left(t\right)dt\;$$

where *C*_0_ is the longitudinal elastic wave velocity in the incident bar. $\varepsilon_{c}\left(t\right)$is the compression strain in the flange. In practice, the calculated *u_s_* will be further optimized by a few trials.

The incident strain $\varepsilon_{i}\left(t\right)$, reflected strain $\varepsilon_{r}\left(t\right)$ and transmitted strain $\varepsilon_{t}\left(t\right)$ are recorded as functions of time *t* using strain gages G_1_ and G_2_ attached to the bars at two locations. The axial forces and displacements on the front and rear ends of the specimen can be calculated from $\varepsilon_{i}\left(t\right)$, $\varepsilon_{r}\left(t\right)$ and $\varepsilon_{t}\left(t\right)$ through the following expressions:

(2) $$F_{1}\left(t\right)=EA\left(\varepsilon_{i}\left(t\right)+\varepsilon_{r}\left(t\right)\right)\;F_{2}\left(t\right)=EA\varepsilon_{t}\left(t\right)$$

(3) $$u_{1}\left(t\right)=C_{0}\int_{0}^{t}\left(\varepsilon_{i}\left(t\right)-\varepsilon_{r}\left(t\right)\right)dt\;u_{2}\left(t\right)=C_{0}\int_{0}^{t}\varepsilon_{t}\left(t\right)dt\;$$

where *F*_1_(*t*) and *F*_2_(*t*) are the forces and *u*_1_(*t*) and *u*_2_(*t*) are the displacements on the front end (right end of the incident bar) and rear end (left end of the transmitted bar) of the specimen, respectively. *E* and *A* are Young’s modulus and cross-sectional area of the bars. *C*_0_ is the longitudinal wave velocity in the bars.

The engineering stress $\sigma_{s}\left(t\right)$, strain $\varepsilon_{s}\left(t\right)$ and strain rate ${\dot{\varepsilon }}_{s}\left(t\right)$ in the specimen can further be obtained as:

(4) $$\sigma_{s}\left(t\right)=\frac{F_{1}\left(t\right)+F_{2}\left(t\right)}{2A_{s}}=\frac{EA}{2A_{s}}\left(\varepsilon_{i}\left(t\right)+\varepsilon_{r}\left(t\right)+\varepsilon_{t}\left(t\right)\right)\;$$

(5) $$\varepsilon_{s}\left(t\right)=\frac{u_{1}\left(t\right)-u_{2}\left(t\right)}{l_{s}}=\frac{C_{0}}{l_{s}}\int_{0}^{t}\left(\varepsilon_{i}\left(t\right)-\varepsilon_{r}\left(t\right)-\varepsilon_{t}\left(t\right)\right)dt$$

(6) $${\dot{\varepsilon }}_{s}\left(t\right)=\frac{C_{0}}{l_{s}}\left(\varepsilon_{i}\left(t\right)-\varepsilon_{r}\left(t\right)-\varepsilon_{t}\left(t\right)\right)$$

where *A_s_* and *l_s_* are the cross-sectional area and gage length of the specimen, respectively.

The principle of the SHTB test assumes that there exists a state of stress equilibrium and uniform deformation in the specimen during the process of impact loading. Therefore, as stress equilibrium is achieved during the test. The forces on the two ends of specimen are equal, *i.e.*, *F*_1_(*t*) = *F*_2_(*t*). The relation $\varepsilon_{i}\left(t\right)+\varepsilon_{r}\left(t\right)=\varepsilon_{t}\left(t\right)$ can be consequently deduced and the engineering stress $\sigma_{s}\left(t\right)$, strain $\varepsilon_{s}\left(t\right)$ and strain rate ${\dot{\varepsilon }}_{s}\left(t\right)$ in the specimen can further be given as:

(7) $$\sigma_{s}\left(t\right)\;=\frac{EA}{A_{s}}\varepsilon_{t}\left(t\right)$$

(8) $$\varepsilon_{s}\left(t\right)=-\frac{2C_{0}}{l_{s}}\int_{0}^{t}\varepsilon_{r}\left(t\right)dt$$

(9) $${\dot{\varepsilon }}_{s}\left(t\right)=-\frac{2C_{0}}{l_{s}}\varepsilon_{r}\left(t\right)$$

Then, the true stress and true strain in the specimen can be easily calculated from the engineering stress and engineering strain using the volume constancy law.

### 2.3. High-Speed Photography

A Photron SAX2 high-speed CCD camera, capable of acquiring images at a frame rate of one million frames per second, is used to photograph the dynamic deformation of the tensile specimens. For better illumination, high performance strobes are placed behind the specimen for silhouette lighting of the specimen. The camera is programmed to record a sequence of 30 separate images at prescribed time intervals, and images are acquired from a point of view normal to the sample.

### 2.4. Quasi-Static Uniaxial Tension Tests

**Figure 3:**
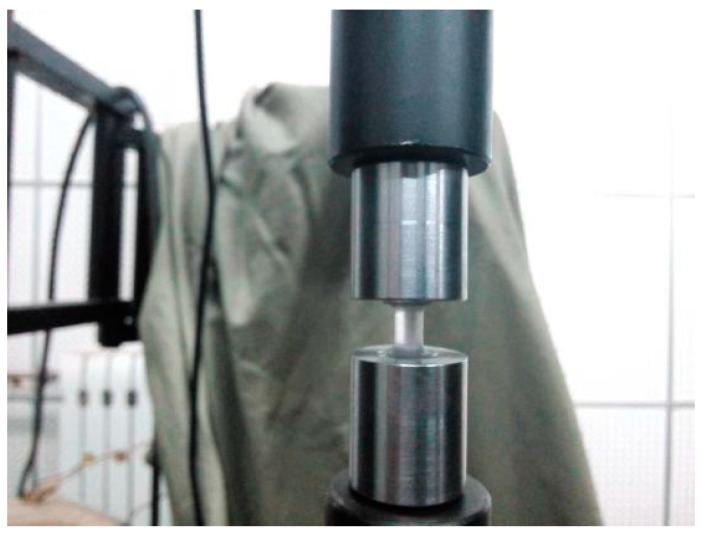
The fixture device connecting the specimen in quasi-static tension tests.

Quasi-static tension tests at strain rates of 0.5 × 10^−3^, 1.0 × 10^−3^, 1.0 × 10^−2^ and 1.0 × 10^−1^ s^−1^ are performed on a DNS-100 electronic universal testing system. The specimen geometry used in quasi-static tension tests is exactly the same as that used in the high-rate tests. A fixture device including two threaded grip made of heat-treated high strength steel is used to connect the specimen, as shown in Figure 3.

## 3. Experimental Results

**Figure 4:**
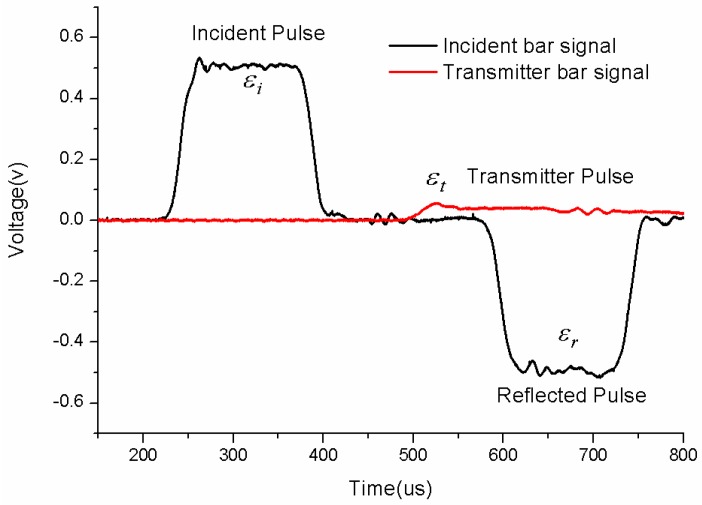
Typical signals measured from the gages on the bars.

**Figure 5:**
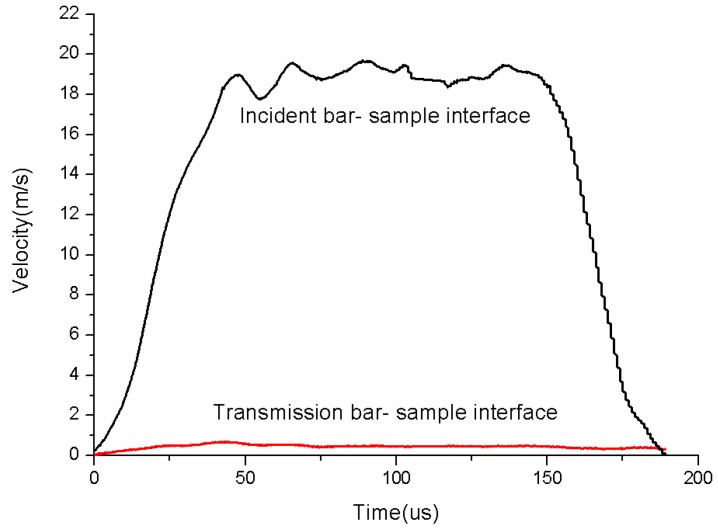
Velocity profiles of the incident bar–specimen interface and transmission bar–specimen interface.

The typical strain gage signals recorded on the incident and transmitted bars are displayed in Figure 4. Obviously, there exists a nearly flat plateau part in the reflected pulse, which means a steady strain rate condition. In addition, the velocity profiles of the incident bar–specimen interface and transmission bar–specimen interface during a test are shown in Figure 5.

**Figure 6:**
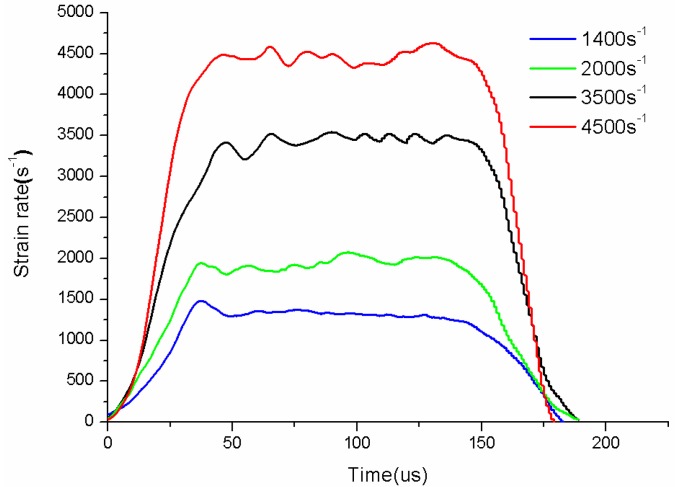
The fixture device connecting the specimen in quasi-static tension tests.

Dynamic tensile tests are performed at strain rates of 1400, 2000, 3500 and 4500 s^−1^. Figure 6 show different strain rates of specimen during tests, we can see the strain rates are stable enough to guarantee the accuracy of the experiments.

**Figure 7:**
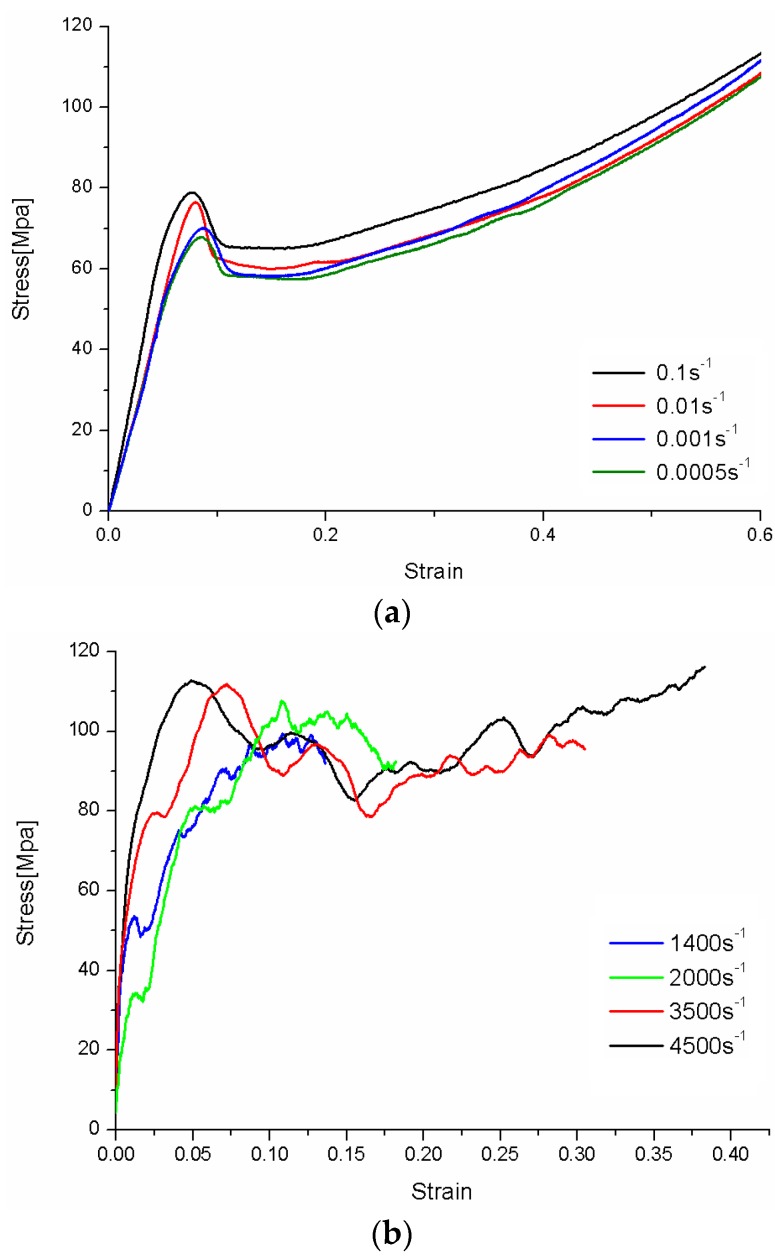
True tensile stress–true strain laws of PC: (**a**) quasi-static; and (**b**) dynamic.

Figure 7 presents the tensile true stress–true strain responses of PC for various strain rates ranging from quasi-static to dynamic loadings. It can be observed for all strain rates that a stress drop occurs following a plastic flow platform when the stress reaches the peak point. The response of PC is strongly dependent on the strain rate. The dynamic response of PC is significantly distinctive from the quasi-static one. In the case of quasi-static loading, the PC specimens are deformed in a ductile manner up to a strain of 0.60. The response includes linear elastic and nonlinear elastic homogeneous deformations and the strain softening and hardening. Furthermore, the yield stress and flow stress increase slightly with the increase of strain rate. In the case of high strain rate loading, the values of yield stress and strain at yield at high strain rates increase apparently than those under quasi-static loading, but they change slightly at different high strain rates investigated in the present paper. Compared with Figure 7a, there are more fluctuations in the dynamic tensile stress–strain curves, which is induced by the imperceptible gaps exist in the threaded connection of bars and specimen. This phenomenon is in agreement with the work of Boyce *et al.* [17]. Here, it should be mentioned that the hardening behaviors are not captured by the loading cases at strain rates of 1400 and 2000 s^−1^ (as shown in Figure 7). This is due to the limit of the split Hopkinson bar measurement in our experimental set-up. In our experiment, the duration of the incident stress pulse is restricted by the length of the prefixed steel bar. Since the duration of the incident stress pulse is constant in all the high rate tests, the total measurable uniaxial strain is decreased with the reduction of strain rate. Therefore, for strain rates of 1400 and 2000 s^−1^, only the softening behaviors are recorded. The hardening behavior after softening cannot be captured.

**Figure 8:**
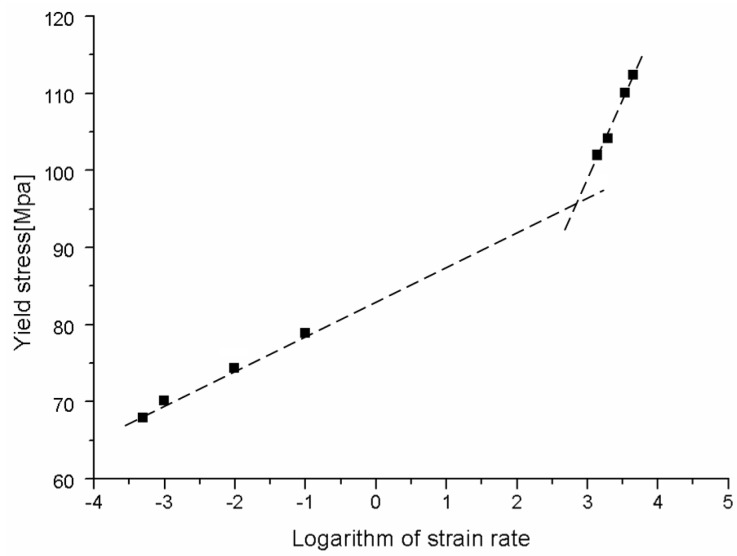
Yield stress of PC over a wide range of strain rates.

For a more comprehensive understanding of the influence of strain rate on the tension properties of PC, the true yield stress plotted against the logarithm of strain rate is shown in Figure 8. It is found that the yield stress of PC is quite sensitive to the strain rate. The yield stress increases linearly with the logarithm of strain rate at both quasi-static regime and high strain rate regime. However, the values of yield stress at dynamic loading conditions are much higher compared with quasi-static results. The experimental results indicate a bilinear relationship between the tensile yield stress and the logarithm of strain rate: at the low strain rates, PC displays weak strain rate dependence, while at high strain rates, the yield stress increases dramatically with increasing strain rate. This phenomenon is similar with the strain rate dependence of compressive yield stress of PC reported by Richetona *et al.* [9]. In addition, Richetona *et al.* [9] indicated that the increase of the compressive yield stress is correlated to secondary molecular processes. An increasing strain rate would decrease the molecular mobility of the polymer chains by making the chains stiffer. This can be also used to explain the strain rate dependence of tensile yield stress of PC.

Note that since the tension tests at strain rate range of 0.1–1000 s^−1^ (moderate rate) are not performed in this study, there is a gap in data about strain rate passing from a logarithm of strain rate of −1 to 3 (see Figure 8). A moderate strain-rate testing set-up is further needed for these tests. At present, we only carried out the quasi-static (below 0.1 s^−1^) and high-rate (exceeding 1000 s^−1^) tension tests. However, the bilinear relationship between the yield stress and the strain rate indicated by our experiment is similar with the strain rate dependence of compressive yield stress of PC reported in the literature. Of course, more experimental data could generate a more precise description of the strain rate dependence of tensile yield stress. In our future study, the moderate strain-rate tests will be employed to investigate the tension behavior of PC in detail.

**Figure 9:**
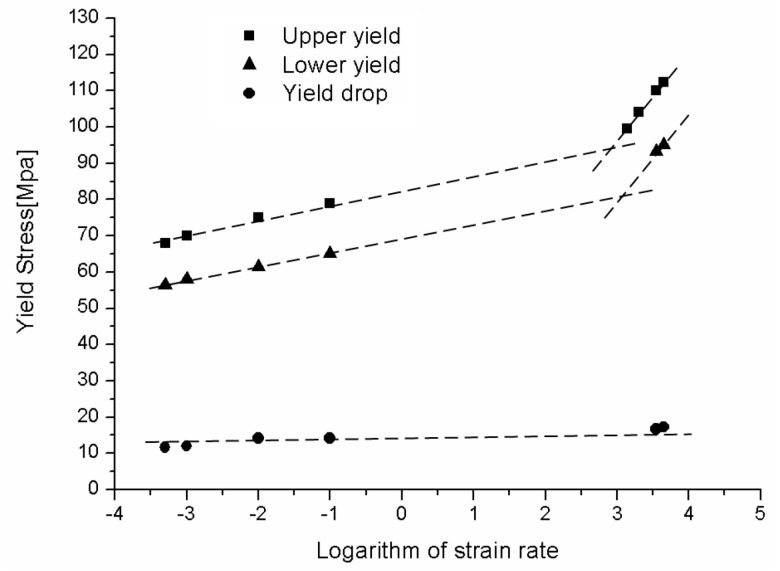
Strain softening behavior of PC for different strain rates.

Figure 9 shows the strain softening behavior of PC from static loading to dynamic loading. Strain softening is an intrinsic mechanical characteristic of amorphous polymers, which demonstrates a dramatic stress drop after yielding. This phenomenon is considered as a degradation of material caused by the rupture of molecular chains network. The upper yield point and lower yield point represent the yield strength and the minimal value after yield, respectively. The yield drop is defined as the difference between upper yield stress and lower yield stress. Although both upper yield stress and lower yield stress increase in bilinear relationship (as shown in Figure 9) with the logarithmic strain rate, the yield drop remains constant, indicating that strain softening is independent on strain rate. It should be noticed that due to the limitation of the measurable uniaxial strains for the tests at strain rates from 1400 to 2000 s^−1^, the lower yield points are not given in Figure 9 for these loading cases.

## 4. Constitutive Model

A constitutive model is essential for the modeling and prediction of the deformation and failure of structures and materials. In this study, the experimental tension stress–strain responses at various strain rates show a nonlinear deformation behavior including elasticity, yield, strain softening and strain hardening. In addition, the experimental investigations demonstrate bilinear relationship between yield stress and the logarithm of strain rate. The similar deformation behavior can be also found for PC under compression loading conditions. In our previous study [23], within the framework of the thermodynamics of irreversible processes, a physically based constitutive model for the high rate behavior of PC was developed based on the uniaxial compression tests at high strain rates. The high rate compressive behavior of PC, including elasticity, yield, strain softening, strain hardening and yield-rate behavior, has been successfully captured by the constitutive model. In this study, this model is extended to describe the strain rate dependent tension response of PC.

### 4.1. Strain Rate Dependent Yield

To model the strain rate dependent yield behavior over the whole strain rate range, a power exponent function is employed here to approximate the bilinear relationship:

(10) $$\sigma_{y}=K\left(1+C_{r}{\dot{\bar{\varepsilon }}}_{eq}^{m}\right)$$

where *K* is the yield stress under uniaxial static loading (minimum strain rate in this study). *C_r_* and *m* are strain rate relative constants. ${\dot{\bar{\varepsilon }}}_{eq}$ is the equivalent strain rate and defined as:

(11) $${\dot{\bar{\varepsilon }}}_{eq}=\sqrt{\frac{2}{3}d\varepsilon_{ij}^{\prime }d\varepsilon_{ij}^{\prime }}$$

where $d\varepsilon_{ij}^{\prime }$ is the deviator strain increment.

### 4.2. Strain Softening and Hardening

The plastic flow of PC consists of strain softening and strain hardening. These two phenomena are caused by different micro-mechanisms. Strain softening is mainly due to the fracture of molecular main chains and the disentanglement of branches, which can be regarded as the damage of molecular network [24]. Meanwhile, strain hardening results from the orientation of main chains along the applied stress direction. In fact, the orientation of main chains and the disentanglement of branches evolve simultaneously. Hence, it is believed that this competitive mechanism between hardening and softening determines the plastic flow of amorphous polymers. In this study, a hardening function combined with the damage evolution is proposed to describe the plastic flow rule of PC.

The hardening function of the virgin material (without damage) can be approximated by a concise function:

(12) $$\sigma_{eff}=C_{h}{\left({\bar{\varepsilon }}^{p}\right)}^{\gamma }$$

where *C_h_* and γ are hardening constants identified by experimental results. $\sigma_{eff}$ is the effective stress of the virgin material. ${\bar{\varepsilon }}^{p}$ is accumulated plastic strain given by:

(13) $${\bar{\varepsilon }}^{p}=\int_{0}^{t}\sqrt{\frac{2}{3}d\varepsilon_{ij}^{p}d\varepsilon_{ij}^{p}}dt$$

where $d\varepsilon_{ij}^{p}$ is the plastic strain increment.

For the purpose of measuring damage macroscopically, Lemaitre [25] proposed an important hypothesis indicating that the deformation of damaged material can be modeled in the form of the constitutive relation of virgin material. In a damaged material, the equivalent stress ${\bar{\sigma }}_{eq}$ can be expressed as a function of damage variable *D* and effective stress $\sigma_{eff}$ of the virgin material:

(14) $${\bar{\sigma }}_{eq}=\left(1-D\right)\sigma_{eff}$$

The damage variable *D* evolves according to the following equation [25]:

(15) $$D=C_{d}{\left({\bar{\varepsilon }}^{p}\right)}^{\chi }$$

where *C_d_* and χ are damage evolution parameters. According to [25], the evolution of damage can be reflected by the decrease of elastic modulus at macroscopic scale, which is easily obtained by cyclic loading/unloading uniaxial test.

Combining Equations (12)–(15), the equivalent stress $\bar{\sigma }$ of a damaged material can be expressed as:

(16) $${\bar{\sigma }}_{eq}=\left(1-C_{d}{\left(\int_{0}^{t}\sqrt{\frac{2}{3}d\varepsilon_{ij}^{p}d\varepsilon_{ij}^{p}}dt\right)}^{\chi }\right)\left(C_{h}{\left(\int_{0}^{t}\sqrt{\frac{2}{3}d\varepsilon_{ij}^{p}d\varepsilon_{ij}^{p}}dt\right)}^{\gamma }\right)$$

In this study, Equation (16) is used to describe the plastic flow rule of PC. The first term of this equation describes the strain softening behavior of PC. The second term reflects the strain hardening.

## 5. Finite Element Simulation of Tension Responses

### 5.1. Finite Element Models

The constitutive model is programmed into the explicit finite element program LS-DYNA by employing a user-defined material subroutine (UMAT). Numerical simulations are conducted to study the deformation behavior of PC specimens during quasi-static and dynamic tensile loading.

The material constants and model parameters are determined by experimental measurement and curve fitting. They are listed as follows.

- Material constants
  Elastic modulus: *E* = 2271 MPa
  Poisson’s ratio: 0.4
  Density: ρ = 1190 kg/m^3^
- Model parameters

The model parameters used in the finite element simulation can be divided into three categories: yield parameters, hardening parameters and damage parameters. The identified values of these parameters are

Yield parameters: *K* = 62.04 MPa, *C_r_* = 0.307, *m* = 0.108
Hardening parameters: *C_h_* = 288.1 MPa, γ = 1.994
Damage parameters: *C_d_* = 0.365, χ = 0.399

**Figure 10:**
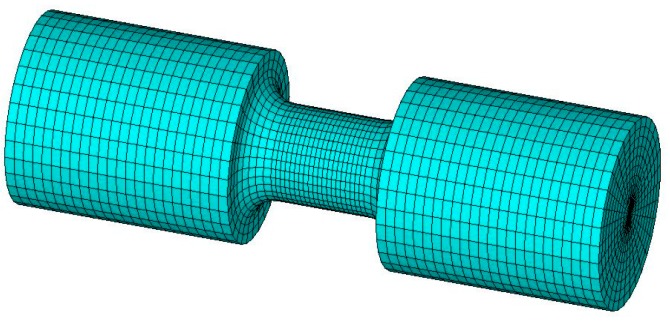
Finite element model of specimen.

The specimen geometry used in the numerical simulations is identical to that in the tensile tests. The specimen is meshed by eight-node brick element, as shown in Figure 10. Since the deformation is mostly concentrated in the gauge section, a finer mesh is chosen for this region. For simplicity, the Hopkinson bars are not included in simulation.

The simulations are performed at constant engineering strain rates corresponding to the actual tension tests. One end of the connection part of the specimen is fixed and the velocity boundary conditions are applied to the other end of the specimen connection part. To keep the consistence with the actual experimental measurement, the engineering stress is defined as the tensile reaction forces divided by the original cross-sectional area in the middle of the specimen. The engineering strain is obtained from the relative displacements between the two ends of the specimen gage section. Finally, the true stress and true strain are calculated from the engineering stress and engineering strain through the volume constancy law.

### 5.2. Numerical Results and Discussions

**Figure 11:**
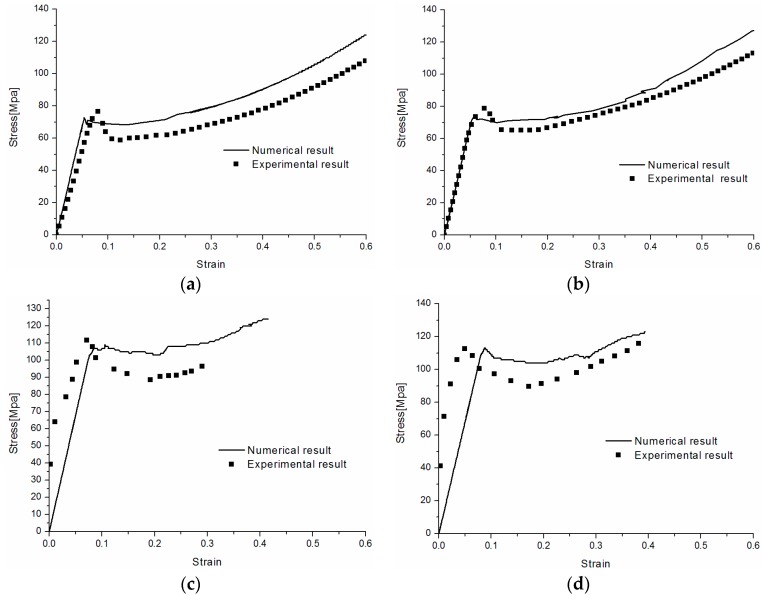
Numerical predictions and experimental results of stress–strain responses of PC at different strain rates: (**a**) 0.01 s^−1^; (**b**) 0.1 s^−1^; (**c**) 3500 s^−1^; and (**d**) 4500 s^−1^.

Firstly, the comparisons of the numerical results of true stress–true strain responses with the experimental data for the uniaxial tension at different strain rates are presented in Figure 11. It can be found that the presented model is capable of capturing the typical behaviors of the tension deformation of PC, including the elastic stage, the yield peak, the post-yield softening and hardening. In addition, the model has proved its ability to describe the rate dependent behavior of PC under tension loading within a wide range of strain rates. 

**Figure 12:**
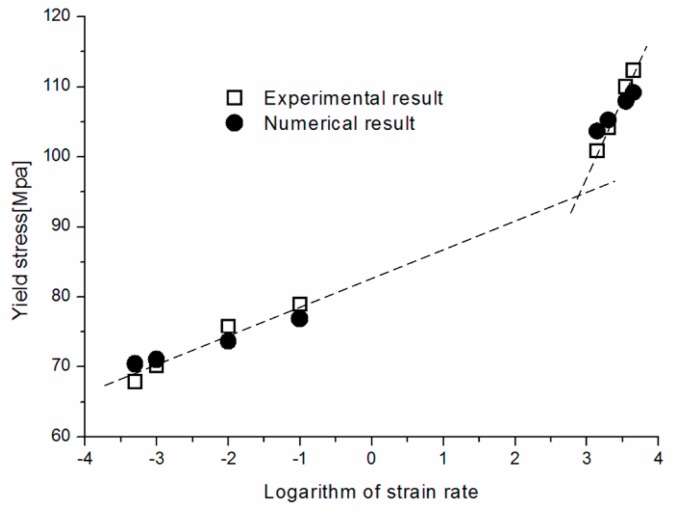
Numerical predictions and experimental results of yield stresses of PC.

Figure 12 shows a comparison of the experimentally observed true yield stress values of PC and the corresponding model predictions. The predicted results coincide well with the experimental data. The yield stress increases with the logarithm of strain rate and shows increased rate sensitivity at high rates.

To clearly understand the mechanics of tension deformation of PC at high strain rate, the numerical results and the high-speed photographs of the gauge region of specimen at various stages of deformation are listed in Table 1. The contours of axial stress at different instants during the deformation at strain rate of 4500 s^−1^ are displayed. The time intervals are carefully chosen to enable a direct comparison with the photographs. It can be observed that to some extent the simulation replicates the deformation profile observed in the photographs. As shown at 20 μs, the displacement initiates from the incident end, resulting in stress fields emanating from that end. After the initial stress wave reverberations, the stress is found to be relatively uniform in the gauge region as shown at 55 μs, which corresponds to elastic deformation. As observed in the photographs from 20 to 55 μs, the gauge uniformly elongates. Yielding then occurs at a stress level of nearly 115 MPa at 65 μs. A neck is found to initiate at the middle region of gauge in the photograph of 65 μs. The stress levels are then higher in the neck region due to the reduced cross-sectional area.

## 6. Conclusions

The tension behaviors of PC at high strain rates up to 4500 s^−^^1^ are investigated in this paper. A comprehensive experimental and finite element study of PC is conducted to investigate the mechanics of deformations within a wide range of strain rates of high-rate tension. The high strain rate tension tests are carried out using a modified SHTB. The experimental tension stress–strain responses at various strain rates show a nonlinear deformation behavior including elasticity, yield, strain softening and strain hardening. In addition, the experimental results indicate that the tension behavior of PC is sensitive to strain rate and the values of yield stress increase dramatically with the increase of strain rate. Based on the experimental observations, a physically based constitutive model is developed and combined with finite element simulations to describe the tension response of PC. The numerical results show that the developed model is capable of capturing the typical behaviors of the tension deformation of PC and describing the rate dependent behavior of PC under tension loading within a wide range of strain rates. Moreover, the numerical results of deformation evolutions at strain rate of 4500 s^−^^1^ are compared with the high-speed photographs. It is shown that the simulation replicates the deformation profile observed in the photographs. The computed axial stresses and the high-speed photographs are used to discuss the stress and deformation evolution of PC during a high rate tension.

## Figures and Tables

**Table 1:** Numerical results and the high-speed photographs during the test at strain rate of 4500 s^−^^1^.

Time (μs)	The contours of axial stress (MPa)		Photograph of the gauge region
20	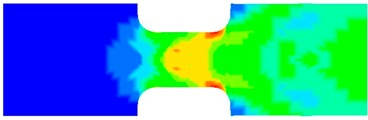	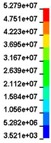	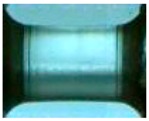
30	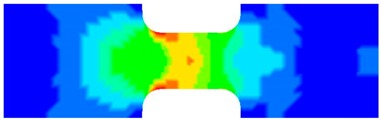	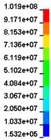	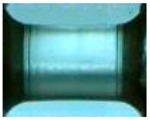
55	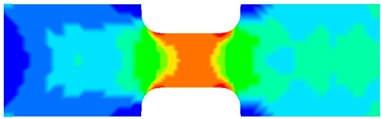	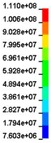	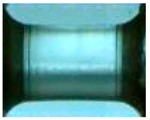
65	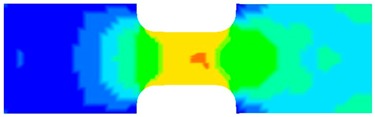	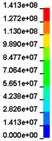	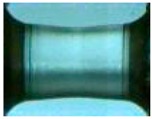
75	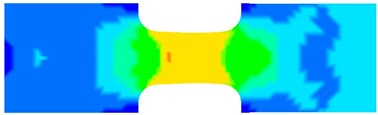	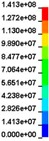	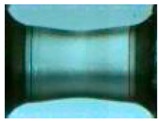
100	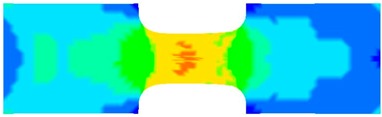	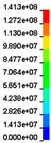	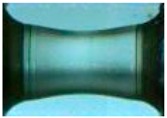
120	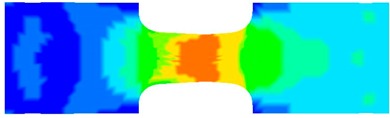	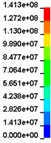	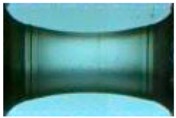
140